# Necrotizing Infection of the Breast: A Case Report on a Rare Presentation of Breast Carcinoma

**DOI:** 10.7759/cureus.24504

**Published:** 2022-04-26

**Authors:** Javeria Tariq, Kulsoom Fatima, Muhammad Usman Tariq, Sana Zeeshan

**Affiliations:** 1 Surgery, Aga Khan University, Karachi, PAK; 2 Radiology, Aga Khan University Hospital, Karachi, PAK; 3 Histopathology, Prince Faisal Cancer Centre, King Fahd Specialist Hospital, Buraydah, SAU; 4 Breast Surgery, Aga Khan University Hospital, Karachi, PAK

**Keywords:** homeopathy, mastectomy, polymicrobial infection, breast carcinoma, necrotizing infections, necrotizing fasciitis

## Abstract

Necrotizing infection (NI) of the breast associated with underlying malignancy is a rare phenomenon characterized by necrosis of breast parenchyma, causing a delay in diagnosis and even leading to sepsis. We present a case of a 42-year-old female with NI of the right breast while on homeopathic treatment for a right breast lump for six months. Tissue culture showed a polymicrobial infection and histopathology established the diagnosis of breast carcinoma. After treating the NI, her breast cancer was managed as per standard guidelines.

## Introduction

A necrotizing infection (NI) of soft tissue may involve the dermis, subcutaneous tissue, fascia, and at times the underlying muscle [1]. The infection usually follows an injury as a result of trauma [2], allowing bacterial entry and the associated inflammation of fascial layers [3]. These infections, although not very common, progress rapidly and are very lethal, usually presenting with a high risk of morbidity and mortality (25%) [3]. The most commonly affected sites include the extremities, perineum, and abdominal wall, with the breast being an extremely rare site of necrotizing fasciitis with only a few case reports published in the literature [1,4].

NI is often categorized into two subtypes: Type 1 is polymicrobial and the more common form, where many different microbes (gram-positive, gram-negative, aerobes, and anaerobes) such as Staphylococcus aureus and Escherichia coli infect together; while Type 2 is a less common but more severe and aggressive form usually involving Group A beta-hemolytic Streptococcus and/or Staphylococcus aureus as the causative agents [2,5].

NI of the breast can often be mistaken for cellulitis or an abscess since the initial symptoms, such as erythema of the skin, mimic cellulitis, thereby leading to potential delays in therapeutic interventions and an associated increase in mortality [3,4]. Therefore, early diagnosis, prompt administration of broad-spectrum antibiotics, and surgical debridement are important to ensure improved outcomes [2,6].

We present a case of a 42-year-old woman with NI of the right breast who was found to have underlying breast carcinoma on diagnostic workup.

## Case presentation

A 42-year-old, married, nulliparous, diabetic, hypertensive female, who was a non-smoker, presented to the emergency room with subjective complaints of fever (not checked by thermometer), severe pain, and foul-smelling bloody discharge from her right breast for two weeks. She had a history of a right breast lump for six months, for which she had been taking some oral homeopathic tablets, the names of which were not recorded. On examination, she had a blood pressure of 132/76 mmHg, pulse of 84 bpm, temperature of 99 °F, and respiratory rate of 14 breaths per minute. The right breast was tender and hard, with a 4 x 3-cm necrotic skin patch on the upper half with bleeding and a palpable right axillary lymph node. The rest of the examination was unremarkable.

**Figure 1:**
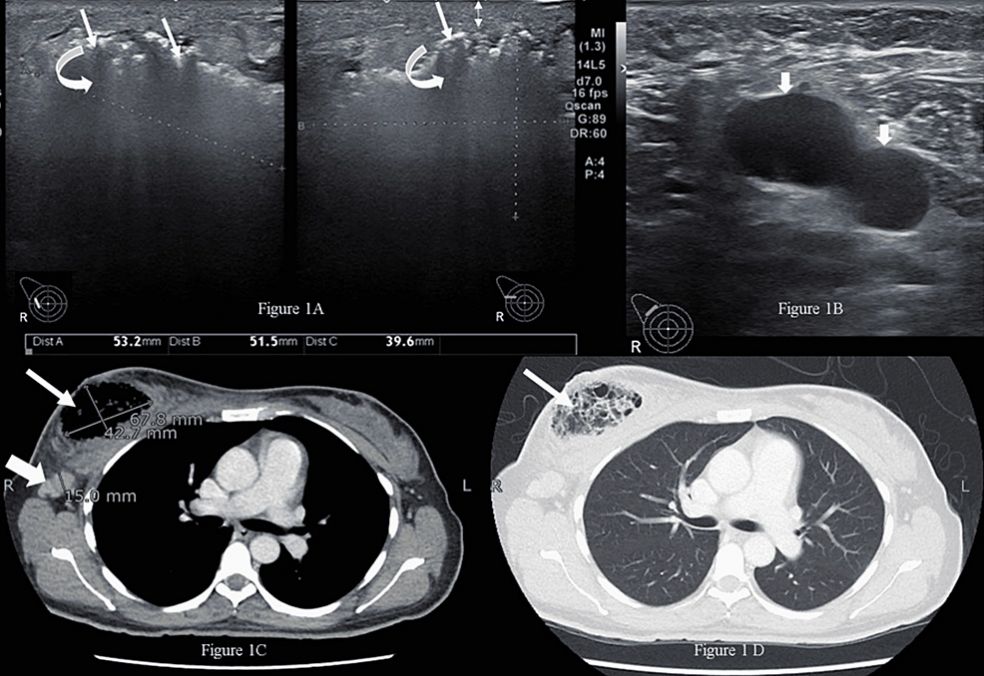
Radiological appearance of the breast lesion (A) Ultrasound image of the right breast upper outer quadrant shows an ill-defined area with echogenic foci representing air (straight arrows) causing dirty shadowing (curved arrows). Skin thickening was also noted (double arrow) (B) Ultrasound of the right axilla shows enlarged hypoechoic lymph nodes (short arrows) with absent hilum (C) Axial contrast-enhanced CT image (mediastinal window) shows the air-containing cavity in the right breast (long thin arrow) and the enlarged right axillary lymph node (short thick arrow) (D) Axial CT image, lung window, at the same level, shows the air-containing right breast cavity with thin internal septations (arrow) CT: computed tomography

The patient's workup showed hyponatremia, uncontrolled blood sugar, and a total leukocyte count of 18,300 cells per cubic millimeter with a neutrophilic shift. Initially, an ultrasound of both breasts was carried out. At the site of hardness in the right breast, there was an ill-defined area showing multiple echogenic foci with dirty posterior shadowing, suggestive of air specks obscuring underlying details (Figure 1A). The area approximately measured 53.2 x 51.5 x 39.6 mm. There was also overlying skin thickening and soft-tissue edema in the upper half of the right breast. No discrete solid lesion was identified. Multiple, abnormal-looking, enlarged hypoechoic lymph nodes were observed in the right axilla showing loss of hilum (Figure 1B). Left breast ultrasound and mammogram were unremarkable. A right mammogram was not performed as the patient had severe pain in her breast. Due to a history of a long-standing lump, there was a high suspicion of underlying malignancy. MRI scan of breasts is considered a standard/recommended imaging procedure and a request for performing an MRI scan was made. However, due to some technical issues in the radiology facility of our institute, an early MRI scan could not be performed. The distressful condition of the patient did not allow us to wait for a late MRI scan appointment or refer the patient to any outside radiology facility. The patient was, therefore, advised to undergo a metastatic workup in the emergency room, which included a contrast-enhanced CT (CECT) of the chest, abdomen, and pelvis, as well as a bone scan. The CT confirmed the presence of an air-filled cavity in the right breast with thin septations and enlarged right axillary lymph nodes (Figures 1C, 1D); however, there was no enhancing mass to suggest neoplasm in either breast. The CT and bone scans were negative for metastasis. The presence of severely tender breast on clinical examination and air within the breast on ultrasound suggested the possibility of NI, which warranted an early surgical intervention to prevent impending sepsis.

**Figure 2:**
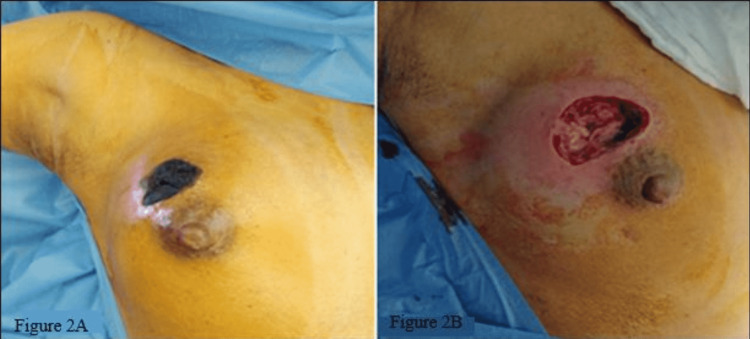
Clinical and perioperative appearance of the wound (A) Appearance of the right breast with necrotic skin patch at 12 o’clock prior to debridement (B) Appearance of the wound after debridement

Given the clinical diagnosis of right-breast NI, the patient underwent debridement (Figures 2A, 2B) and a biopsy of the right breast tissue. Intraoperatively, necrotic, foul-smelling tissue with air bubbles was noticed with healthy pectoralis major muscle. Necrotic tissue was debrided followed by Edinburgh University Solution Of Lime (EUSOL) dressing. The patient was discharged on clindamycin, metronidazole, ciprofloxacin, and daily dressings. An ultrasound-guided right axillary lymph node biopsy was also advised.

**Figure 3:**
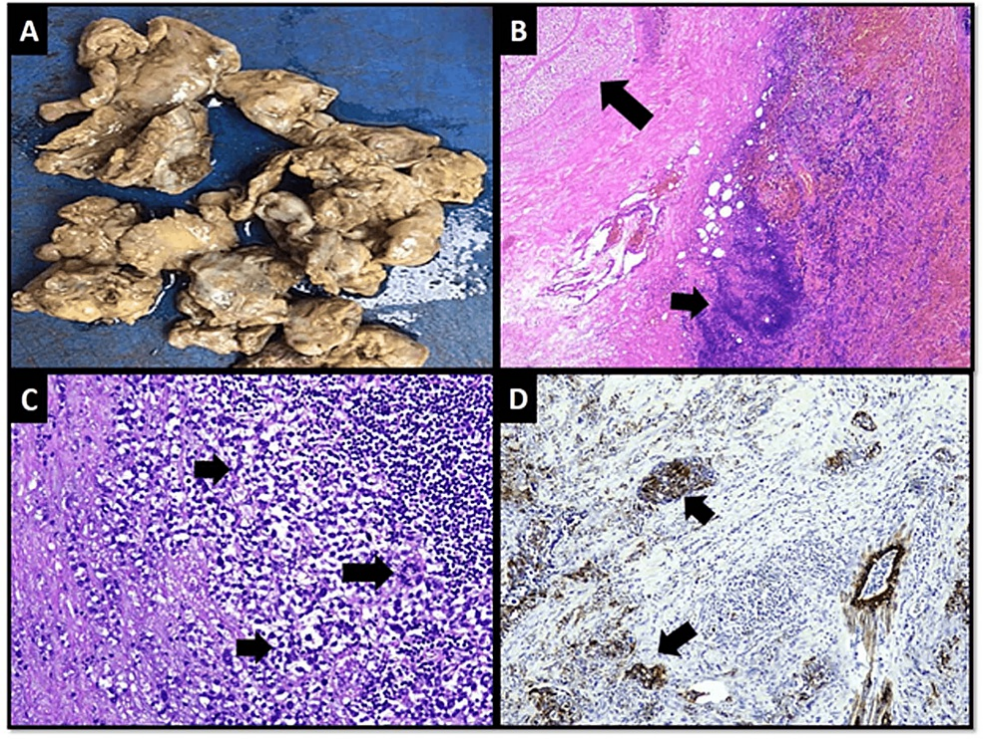
Gross and microscopic features of the wound debridement specimen (A) Gross appearance of necrotic debrided tissue (B) Low-power microscopic view showing areas of necrosis (long arrow) and dense inflammation (short arrow) (C) High-power view of tumor cells arranged in nests and trabeculae (short arrows). Mitotic activity is easily appreciable (long arrow) (D) Cytokeratin CAM5.2 IHC stain highlighting tumor cells

The debrided tissue was tan-brown in color, soft, and friable (Figure 3A). Microscopic examination of the debrided tissue revealed an invasive breast carcinoma of no special type [invasive ductal carcinoma (IDC), NST grade III] along with extensive necrosis and dense acute and chronic inflammation (Figures 3B, 3C). Cytokeratin and epithelial membrane antigen (EMA) immunohistochemical (IHC) stains highlighted nests and clusters of tumor cells masked by a dense inflammatory infiltrate (Figure 3D). Tumor cells demonstrated positivity for E-cadherin and negative expression for synaptophysin and p63 IHC stains. Estrogen, progesterone receptors, and Her2/neu were negative. The right axillary node biopsy was positive for nodal metastasis, and the patient was staged as cT4N1MO. A tissue culture showed a few colonies of Staphylococcus aureus and Enterococcus species suggestive of NI. After a discussion at a multidisciplinary tumor board meeting, the patient underwent a right modified radical mastectomy. Her postoperative course was unremarkable.

**Figure 4:**
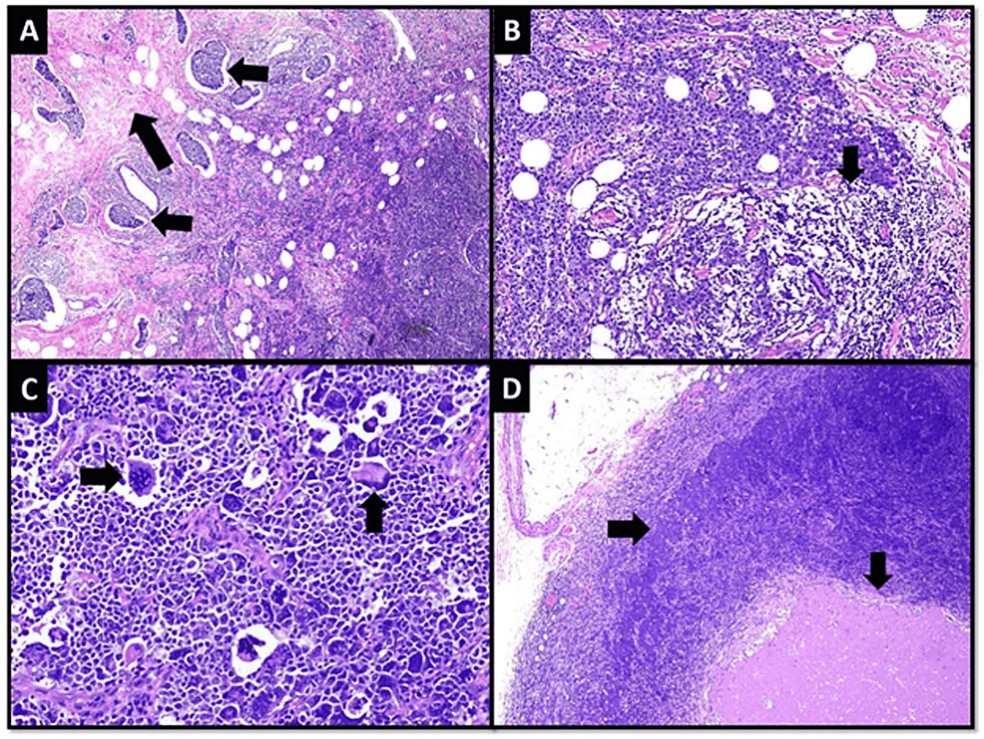
Microscopic features of the tumor in MRM specimen (A) Low-power microscopic view of the tumor showing fibrosis (long arrow) and extensive lymphovascular invasion (short arrows) at the periphery (B) Lymphoid aggregates are also seen along tumor nests (arrow) (C) High-power view of tumor cells showing marked nuclear pleomorphism (vertical arrow) and brisk mitotic activity (horizontal arrow) (D) Lymph node showing metastatic carcinoma (horizontal arrow) and necrosis (vertical arrow) MRM: modified radical mastectomy

A macroscopic examination of the mastectomy specimen revealed an infiltrating lesion in the upper half with ulcerated overlying skin. Microscopic examination revealed invasive breast carcinoma (IBC), NST grade III. Marked fibrosis and extensive lymphovascular invasion were observed at the tumor’s periphery (Figure 4A). The extent and intensity of inflammation had reduced markedly but patches of lymphoid aggregates were still present along tumor cells (Figure 4B). Tumor cells were markedly pleomorphic and showed brisk mitotic activity (Figure 4C). Eight out of 17 lymph nodes showed tumor metastasis along with necrosis (pT4bN2a) (Figure 4D).

The patient is currently under the treatment of medical oncology services for adjuvant chemotherapy, which will be followed by radiation to the right chest wall, axilla, and supra-clavicular nodes.

## Discussion

NI of the breast is a rare but potentially lethal bacterial infection. It is characterized by necrosis of the subcutaneous tissue and breast parenchyma due to the production of bacterial toxins that can even lead to sepsis and systemic toxicity [7].

Our patient was a known case of diabetes mellitus, and conditions like diabetes, alcoholism, peripheral vascular disease, chronic renal failure, malignancy, or immunosuppression are known to increase the risk of necrotizing breast infections [6]. The majority of the cases occur after trauma or breast intervention/surgery while about one-fifth of the cases are idiopathic [8]. We came across only one recently reported case with an underlying breast cancer similar to our case [9]. Existing literature suggests that misdiagnosis of NI of the breast is quite common, as it is often mistaken for cellulitis, breast abscess, mastitis, or inflammatory breast carcinoma [7]. Usually, NI of the breast is unilateral [4], as in our case. Clinically, the patients present with erythema, swelling, induration, severe pain that is disproportionate to local findings, and edema followed by bullae formation containing serous fluid [7,10]. A study by Giuliano et al. demonstrated the presence of facultative and anaerobic organisms in 77% of the patients with NI [11,12]. Our patient also presented with a foul-smelling bloody discharge, severe pain, and edema, with tissue culture positive for Staphylococcus aureus and Enterococcus species. The presence of gas-forming organisms can be detected through crepitation on palpation [12,13]. Our patient showed extensive air on imaging, suggestive of the presence of organisms responsible for necrosis.

It has been observed that some patients who discover a breast lump usually delay in seeking healthcare due to the fear of surgery or cancer, while others resort to alternative therapeutic options, especially homeopathic treatment [14]. Likewise, our patient also opted for homeopathy for six months on a family member’s advice. Homeopathy is not an uncommon approach to cancer management, with 24% of females resorting to homeopathic treatment for breast cancer [15]. Our patient opted for homeopathy as the sole early treatment, which led to a delay in the diagnosis of the underlying breast malignancy, and we propose that homeopathic treatment might have played a role in precipitating NI and, hence, worsening the underlying condition.

It is important to differentiate NI from cellulitis or breast abscesses, considering a similar early presentation, and thus avoid delays in treatment [13]. While breast abscesses are usually treated with ultrasound-guided needle aspirations [13], the management of NI involves early surgical referral of patients, prompt administration of broad-spectrum antibiotics to ensure coverage of polymicrobial NI, and an urgent gram stain or culture followed by surgical debridement [10,12,13,16]. Our patient was initially managed on these lines and then underwent modified radical mastectomy (MRM) due to the underlying malignancy, to be followed by adjuvant chemoradiation.

## Conclusions

NI of the breast is a rare condition that is difficult to diagnose. Being highly fatal, it requires prompt recognition by clinical examination, imaging, and tissue culture followed by management with broad-spectrum antibiotics and debridement, which is the mainstay of therapy. It may mask an underlying malignancy, leading to a delay in diagnosis and progression and resulting in a mastectomy. Alternate therapies for breast cancer, such as homeopathic treatment, are becoming increasingly common due to their low cost and minimal side effects, as shown by some in vitro studies; however, over-the-counter usage of such medicines may lead to further aggravation of the underlying condition. Further research is required to determine the true anti-tumor potential of homeopathy medicines.
